# Autogenous reproduction by *Ornithodoros turicata* (Ixodida: Argasidae) females and vertical transmission of the tick-borne pathogen *Borrelia turicatae* (Spirochaetales: *Borreliaceae*)

**DOI:** 10.1128/aem.01032-23

**Published:** 2023-10-25

**Authors:** Serhii Filatov, Aparna Krishnavajhala, Job E. Lopez

**Affiliations:** 1 Department of Pediatrics, National School of Tropical Medicine, Baylor College of Medicine, Houston, Texas, USA; 2 Department of Molecular Virology and Microbiology, Baylor College of Medicine, Houston, Texas, USA; UMR Processus Infectieux en Milieu Insulaire Tropical, Sainte-Clotilde, France

**Keywords:** relapsing fever, argasid, transovarial transmission, spirochetes, autogeny

## Abstract

*Ornithodoros turicata* is a vector of relapsing fever spirochetes in North America and transmits *Borrelia turicatae* to a variety of vertebrate hosts. The remarkably long lifespan of *O. turicata* and its ability to maintain spirochetes horizontally (between life stages) and vertically (to progeny) promote the perpetuation of *B. turicatae* in nature. Nevertheless, the reproductive biology of *O. turicata* is poorly understood. In this report, we collected ticks from a park within a neighborhood of Austin, TX. They were reared to adulthood, and male ticks were individually housed with females. We observed autogenous reproduction, which is the ability to produce eggs without the need for a blood meal, and further investigated vertical transmission of *B. turicatae* by quantifying filial infection rates in a cohort of progeny ticks. These results indicate that *O. turicata* transovarially transmits *B. turicatae* during autogenous reproduction and further signify the tick as a natural reservoir of the spirochetes.

**IMPORTANCE:**

Previous research has implicated *Ornithodoros* ticks, including *Ornithodoros turicata*, as long-term reservoirs of relapsing fever (RF) spirochetes. Considering the tick’s long lifespan and their efficiency in maintaining and transferring spirochetes within the population, the infection could persist in a given enzootic focus for decades. However, little is known about the relative importance of horizontal and vertical transmission routes in the persistence and evolution of RF *Borrelia*. Our observations on the reproductive biology of *O. turicata* in the absence of vertebrate hosts indicate an additional mechanism by which *Borrelia turicatae* can be maintained in the environment. This work establishes the foundation for studying *O. turicata* reproduction and spirochete-vector interactions, which will aid in devising control measures for *Ornithodoros* ticks and RF spirochetes.

## INTRODUCTION

The soft tick *Ornithodoros turicata* is a major vector of relapsing fever (RF) spirochetes in North America and is commonly found throughout the southern United States into Mexico (1). Wherever the vector occurs, enzootic foci can be formed between the pathogenic spirochete, *Borrelia turicatae,* and their wildlife hosts. Moreover, spillover events occur when humans or companion animals enter habitats infested with infected ticks. For example, recent work indicates that *B. turicatae* is an emerging threat in parks and recreational areas in highly populated cities of Texas (2 – 4). To further understand the public health impact of *B. turicatae*, knowledge of the tick’s biology and pathogen-vector interactions is needed.

The life cycle of *O. turicata* is complex and the dynamics of vertical transmission of *B. turicatae* are poorly understood. The life span of *O. turicata* is nearly 10 years (5), and adult female ticks can oviposit multiple times (6, 7). Davis reported a cohort of field-collected *O. turicata* females oviposited five times in a 12-month period (7). Once eggs hatch, *O. turicata* has upwards of five instars as nymphs (5). After molting into adults, vertically infected ticks can maintain *B. turicatae* for at least five generations (7). Collectively, prior work indicates that infected *O. turicata* can bypass the need to acquire the infection from a vertebrate host and serve as a reservoir for *B. turicatae*.

An interesting nuance of *Ornithodoros* reproduction that should be considered in combination with transovarial transmission is autogeny. This is the ability to produce eggs without the need for a blood meal. While known to occur in *Ornithodoros* ticks, autogeny varies between species (8). Endris and colleagues reported that during 2 years of rearing *Ornithodoros puertoricensis* they failed to observe autogeny (9). Alternatively, autogeny has been reported in species like *Ornithodoros tholozani* and *Ornithodoros fonsecai* (8, 10). However, we have not found a report characterizing autogeny in *O. turicata*.

The current study was based on a serendipitous observation while assessing *O. turicata* reproduction in a group of naturally infected ticks. Interestingly, as the nymphs molted into adults, we observed cases of autogeny. In one cohort of ticks, we determined rates of transovarial transmission to the F1 progeny. Our work indicates that blood feeding in the first gonotrophic cycle is not essential for *O. turicata* female reproduction and that transovarial transmission occurs by autogeny.

## RESULTS

### Collection of ticks from a park in Austin, TX

**Fig 1:**
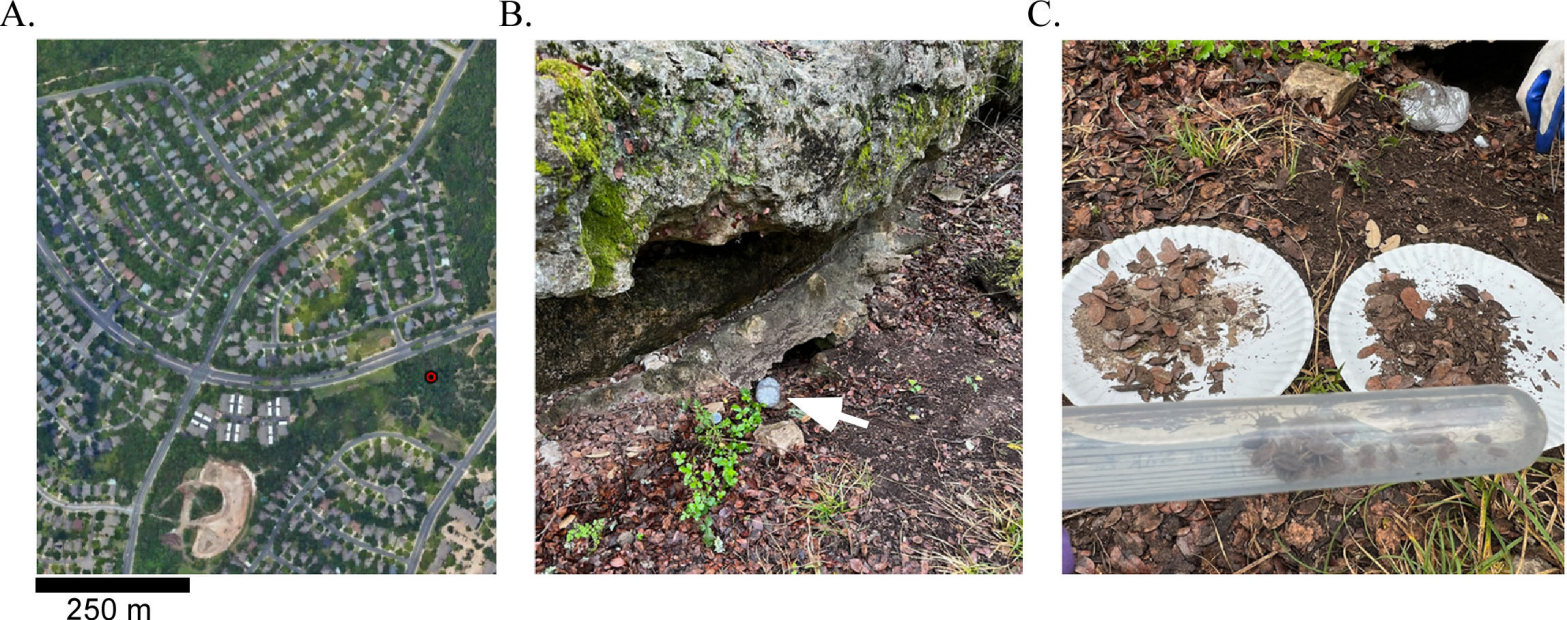
Collection site of *Ornithodoros turicata* in Austin, TX, USA. Shown is an aerial view (Google Earth) of the collection site with a scale in the bottom left corner (**A**). The red dot represents the location where ticks were collected. A container with dry ice was placed by a limestone outcropping and used to bait ticks (**B**). The white arrow points to the dry ice container. Also shown is a 15 mL tube containing the ticks that were collected (**C**). Shown on the paper plates is the material from which the ticks were harvested (**C**).

Tick collections were part of routine surveillance of *Ornithodoros* species in populated cities of Texas. A park was identified in a neighborhood of south Austin (Fig. 1A). The habitat consisted of limestone outcroppings and Live Oak Mesquite Savanna (Fig. 1B). As ticks were lured from outcroppings with dry ice as a source of carbon dioxide, they were collected and housed together (Fig. 1C). We collected 41 ticks. Of these, 15 were adults and 26 were nymphs. Five nymphs died between the time the ticks were collected and evaluated in our laboratory.

### Speciation of field-collected ticks

In the laboratory, we determined the species of field-collected ticks by microscopy and through a molecular approach. Morphological characterization indicated that the ticks were *O. turicata*. For molecular typing, we amplified a portion of the *cox1* gene from two ticks, which produced a ~700 nucleotide fragment, as determined by agarose gel electrophoreses. Sequencing and BLAST analysis determined 99.71% nucleotide identity with *O. turicata*. The sequences were deposited to GenBank under accession numbers OR047917 and OR047918. These findings indicated an additional distribution record of *O. turicata* in Austin, TX.

### Autogenous reproduction by *O. turicata*

Of the 21 live field-collected late-instar nymphs assessed, three died after blood feeding on laboratory mice, nine molted into females, and nine into males. We split the adults into individual tubes housing a male tick with a female, and within 90 days there were numerous larvae in four out of nine tubes. These findings indicated that *O. turicatae* females reproduced and laid eggs autogenously. We subsequently determined whether transovarial transmission occurred in one of the cohorts from a single pair of adults.

### Assessment of transovarial transmission

We reared the larvae to the second-instar nymphs and transmission to susceptible animals was attempted to determine whether these ticks were infected with *B. turicatae*. Feeding F1 second-instar nymphs on mice indicated that the ticks were infected (Table 1). We visualized spirochetes in the blood of five of seven animals by the fifth day after feeding ticks. Spirochetes were observed in the blood of another animal on the ninth day. The mouse in which we failed to detect spirochetes by microscopy seroconverted to *B. turicatae* protein lysates as determined by immunoblotting (Table 1). This indicated that the infection was below the limit of detection by microscopy.

**TABLE 1:** Assessment of murine infection and *B. turicatae* densities in autogenously produced ticks

Mouse #	Assessment of infection		Number of ticks infected/tested^*b*^	$\bar{X}$ (SD) *flaB* copies per infected tick^*b*^
	Microscopy (DPF)^*a*^	Seroconversion by immunoblot		
M1	+(5)	ND^*c*^	3/15	2.28 × 10^5^ (±8.2×10^4^)
M2	−	+	7/15	2.29 × 10^5^ (±9.49×10^4^)
M3	+(5)	ND^*c*^	8/15	2.14 × 10^5^ (±1.05×10^5^)
M4	+(9)	+	5/15	2.3 × 10^5^ (±1.05×10^5^)
M5	+(4)	+	NA^*d*^	NA^*d*^
M6	+(4)	+	NA^*d*^	NA^*d*^
M7	+(4)	+	NA^*d*^	NA^*d*^

^*a*^ DPF: days post tick feeding.

^*b*^ As determined by qPCR.

^*c*^ ND: not determined because the animal was sacrificed for spirochete isolation.

^*d*^ NA: not applicable because the ticks were kept alive for future experiments.

### Rates of filial infection in F1 nymphs

After ticks molted to the third-instar nymph, qPCR determined infection rates in 60 ticks (Table 1; Fig. S1). The remaining ticks were saved for future experiments. Out of the 60 F1 ticks, 23 were positive for *B. turicatae flaB* gene and filial infection rates were 38.3% (95%CI: 26.3%–51.8%). These findings determined vertical transmission rates in the offspring of ticks that autogenously reproduced.

## DISCUSSION

This study arose from laboratory observations of *O. turicata* that were collected in a public park within a neighborhood of Austin, TX. Ticks were reared from the nymphal stage to adulthood, and we observed autogenous reproduction. While autogeny has been reported in a close relative of *O. turicata*, *Ornithodoros parkeri* (8, 11)*,* literature on the former is absent. The reasons for this lack of data are unclear. Without designed experiments housing individual ticks and tracking their molt progress, the phenomenon may have been overlooked during colony maintenance.

In Argasidae, nuances in autogeny depend on reproductive and feeding behaviors. Several species are fully autogenous, such as *Otobius* spp. and *Antricola* spp. In the adult stage, these genera have vestigial mouthparts and never blood feed (12). *Alveonasus lahorensis* females are obligatory autogenous in their first gonotrophic cycle but have to secure a blood meal to produce subsequent egg batches (13). Facultative autogeny in the first gonotrophic cycle has been reported at variable rates in species currently classified in the genera *Ornithodoros*, *Argas*, and *Carios* (8, 10, 12, 14). Interestingly, in other arthropods like mosquitoes, autogeny varies extensively between conspecific populations across distribution ranges (15). While studies suggest that genetic and environmental factors (e.g., temperature) modulate autogeny in soft ticks (11), additional work is needed in *O. turicata* to identify its frequency between geographically distinct populations.

We evaluated filial infection rates and successful *B. turicatae* transmission to mice from the autogenously produced cohort of *O. turicata*. These findings indicated that after the transovarial passage through to the next generation larvae, *B. turicatae* remained infectious. Determining the infectivity of *B. turicatae* to mice was important because prior work on continuous vertical transmission through successive generations of *Ornithodoros* species is ambiguous. For example, ~22% of vertically infected cohorts of *Ornithodoros papillipes* failed to transmit *Borrelia sogdiana* in the eighth generation compared to ~100% in earlier generations (16). Additionally, a complete loss of transmissibility of *Borrelia duttonii* by tick bite was reported by the fifth generation in transovarially infected *Ornithodoros moubata* (17). However, Burgdorfer and Varma failed to reproduce these findings using a different strain of spirochete and population of tick (18). With *B. turicatae*, spirochetes remained infectious to mice after vertical maintenance by the vector for five generations (7). In these previous studies, the life stage where ticks became infected was known to the investigators. A caveat in our work was that *O. turicata* ticks were field collected, and it was unclear if the infected parental tick acquired spirochetes vertically or from an infectious blood meal. We have isolated and cultured the *B. turicatae* strain from this study and will assess vertical transmission after infecting *O. turicata* at different life stages.

We determined spirochete densities in individual ticks to better understand transovarial transmission rates in the offspring of autogenously reproduced ticks. Our estimates of *B. turicatae* loads were based on *flaB* copies per tick and initially appeared high. However, work with *Borrelia hermsii* and *Borrelia burgdorferi* indicates that the pathogens are polyploid having ~16 chromosomal copies per cell depending on the bacteria’s growth stage (19 – 21). With this consideration, a more accurate estimation of *B. turicatae* densities in the ticks was likely ~10-fold less, or ~10^4^ spirochetes per tick.

While our study began to evaluate autogeny and vertical transmission of spirochetes by *O. turicata,* there were limitations. For example, we fed cohorts of larvae or first-instar nymphs on mouse pups. Consequently, a portion of uninfected ticks within the cohort could have acquired *B. turicatae* by co-feeding with infected ticks or through hyperparasitism. Acquisition of RF *Borrelia* by co-feeding has not been reported for *B. turicatae* or other soft tick-borne RF spirochete species, presumably because the ticks are rapid feeders. Additionally, while hyperparasitism has been reported as a route of acquisition of RF spirochetes in *Ornithodoros hermsi* and *Ornithodoros papillipes* (22, 23), in *O. turicata* there are only occasional observations of conspecific ticks parasitizing each other (5, 24). For this study, it was impractical to feed individual larvae on mice, and we are still optimizing an approach to detect *B. turicatae* DNA in individual eggs and larvae.

Our findings are for one strain of *B. turicatae*. It will be important to know the rates of vertical transmission between *B. turicatae* isolates given their genomic plasticity. RF spirochetes possess complex genomes with upwards of 15 plasmids, and isolates vary significantly in their plasmid content (4). Furthermore, in this study, we evaluated one population of ticks from Texas, while *O. turicata* possesses a wide distribution range. More elaborate studies will identify phenotypic differences between spirochete isolates and tick populations.

It is important to define the cost-benefit ratio of autogeny for both the tick vector and pathogen. Tick eggs and larvae are less resistant to adverse environmental conditions (13, 25); however, autogenous reproduction could maximize fitness in newly established tick populations with transient hosts. This is consistent with ecological bet-hedging (26). Indeed, facultative autogeny can accelerate population growth after dispersal into new suitable habitats in patchy landscapes, as has been proposed in triatomine kissing bugs (27). Additionally*,* newly colonized habitats containing *B. turicatae*-infected larvae could facilitate a rapid expansion of enzootic foci. Studies by Davis highlighted the significance of *O. turicata* as a long-term reservoir of *B. turicatae* (7)*,* and our findings support this notion. While more work is needed to delineate the mechanisms of vertical transmission, our work lays the foundation for studying *O. turicata* reproduction and spirochete-vector interactions.

## MATERIALS AND METHODS

### Collection and speciation of ticks

In March 2022, field efforts were implemented to collect ticks. The ticks were baited with dry ice, collected in tubes, and transferred to our lab where they were housed at 24°C ± 2°C and 85% relative humidity (28). The species of tick was confirmed by microscopy and through PCR amplification of a fragment of the mitochondrial *cox1* gene using DNA from two ticks and the LCO and HCO primers (29). The amplicons were purified using the Mag-Bind Total Pure NGS beads (Omega Bio-tek, Norcross, GA, USA) and sequenced. The sequences were deposited in GenBank.

### Tick feedings and detection of murine infection

To check for *Borrelia* infection, field-collected *O. turicata* were divided into pools of 8–10 ticks and fed on Institute for Cancer Research (ICR) mice, as previously described (4). Blood was collected for 10 consecutive days by tail nick, and infection was determined by visualizing spirochetes using a dark field microscope (Zeiss Axio Imager A2, Oberkochen, Baden-Württemberg, Germany). After feeding, the ticks’ life stage was determined by visualization of the genital aperture. They were sorted into pairs of males and females, individual females, or groups of nymphs (genital aperture is not apparent at this developmental stage). Ticks were housed in 50 mL TubeSpin Bioreactor tubes (MidSci, St. Louis, MO, USA).

The F1 progeny from a single female was further evaluated. The remaining larvae from the other females that autogenously reproduced were kept for other studies. For rearing *O. turicata*, we only used unexposed mice; the animals were never recycled to feed ticks. Larvae and first-instar nymphs were reared by feeding them on mouse pups. As second-instar nymphs, ticks were tested for infection by feeding on seven adult ICR mice (15–18 ticks per animal; *n* = 107). Infection was evaluated by microscopy, as stated above. Thirty days after tick feedings, all animals were euthanized and blood was collected to determine infection by evaluating seroconversion to *B. turicatae* protein lysates, as reported (4).

### Molecular detection of *B. turicatae* in F1 ticks

Filial infection rates were determined on a subset of progeny using a duplex qPCR assay targeting *B. turicatae flaB* and the *O. turicata B-actin* gene, as described in reference (30). DNA was extracted from individual unfed third-instar nymphs using the DNeasy Blood and Tissue kit (Qiagen, Hilden, Germany). Each sample was run twice in triplicate. Spirochete loads in each sample were determined as previously described (31). The Wilson interval with continuity correction was calculated for the resulting point estimate of filial infection rates to obtain a 95% confidence interval using R (32).

## SUPPLEMENTAL MATERIAL

Supplemental Figure 1 aem.01032-23-s0001.tif Calculated flaB copies per tick.

ASM does not own the copyrights to Supplemental Material that may be linked to, or accessed through, an article. The authors have granted ASM a non-exclusive, world-wide license to publish the Supplemental Material files. Please contact the corresponding author directly for reuse.

## References

[1] Lopez JE , Krishnavahjala A , Garcia MN , Bermudez S . 2016. Tick-borne relapsing fever spirochetes in the Americas. Vet Sci 3:16. doi:10.3390/vetsci3030016 28959690 PMC5606572

[2] Bissett JD , Ledet S , Krishnavajhala A , Armstrong BA , Klioueva A , Sexton C , Replogle A , Schriefer ME , Lopez JE . 2018. Detection of tickborne relapsing fever spirochete. Emerg Infect Dis 24:2003–2009. doi:10.3201/eid2411.172033 30160650 PMC6199987

[3] Ellis L , Curtis MW , Gunter SM , Lopez JE . 2021. Relapsing fever infection manifesting as aseptic meningitis. Emerg Infect Dis 27:2681–2685. doi:10.3201/eid2710.210189 34546167 PMC8462328

[4] Krishnavajhala A , Armstrong BA , Kneubehl AR , Gunter SM , Piccione J , Kim HJ , Ramirez R , Castro-Arellano I , Roachell W , Teel PD , Lopez JE . 2021. Diversity and distribution of the tick-borne relapsing fever spirochete Borrelia turicatae. PLoS Negl Trop Dis 15:e0009868. doi:10.1371/journal.pntd.0009868 34813588 PMC8651100

[5] Francıs E . 1938. Longevity of the tick Ornithodoros turicata and of Spirochaeta recurrentis within this tick. Public Health Reports (1896-1970) 53:2220. doi:10.2307/4582740

[6] Kemp HA , Moursund W , Wright HE . 1934. Relapsing fever in Texas. IV. Ornithodorus turicata Duges: à vector of the dsease. Am J Trop Med Hyg 14. doi:10.4269/ajtmh.1934.s1-14.479

[7] Davis GE . 1943. Relapsing fever: the tick Ornithodoros turicata as a spirochetal reservoir. Public Health Reports (1896-1970) 58:839. doi:10.2307/4584474

[8] Feldman-Muhsam B . 1973. Autogeny in soft ticks of the genus Ornithodoros (Acari: Argasidae). J Parasitol 59:536. doi:10.2307/3278790

[9] Endris RG , Haslett TM , Monahan MJ , Phillips JG . 1991. Laboratory biology of Ornithodoros (Alectorobius) Puertoricensis (Acari: Argasidae). J Med Entomol 28:49–62. doi:10.1093/jmedent/28.1.49 2033619

[10] Santiago ACC , Duarte LL , Martins TF , Onofrio VC , Nieri-Bastos FA , Pacheco R de C , Melo ALT , Marcili A , Barros-Battesti DM . 2019. Occurrence of autogeny in a population of Ornithodoros fonsecai (Acari: Argasidae). Ticks Tick Borne Dis 10:1078–1084. doi:10.1016/j.ttbdis.2019.05.014 31182378

[11] Pound JM , Oliver JH Jr , Andrews RH . 1984. Effects of temperature and tick weight on expression of autogeny in the argasid tick Ornithodoros parkeri Cooley (Acari: Argasidae). J Parasitol 70:279–284. doi:10.2307/3281876 6470890

[12] Diehl PA , Aeschlimann A , Obenchain F . 1982. Tick reproduction: oogenesis and oviposition, p 277–350. In Physiology of ticks. Elsevier.

[13] Balashov YS. 1967. Blood-sucking ticks (Ixodoidea)-vectors of diseases of man and animals. Nauka, Leningrad.

[14] Chinzei Y , Okuda T , Ando K . 1989. Vitellogenin synthesis and ovarian development in nymphal and newly molted female Ornithodoros moubata (Acari: Argasidae). J Med Entomol 26:30–36. doi:10.1093/jmedent/26.1.30

[15] Arich S , Haba Y , Assaid N , Fritz ML , McBride CS , Weill M , Taki H , Sarih M , Labbé P . 2022. No association between habitat, autogeny and genetics in Moroccan Culex pipiens populations. Parasit Vectors 15:405. doi:10.1186/s13071-022-05469-3 36329500 PMC9635193

[16] Balashov YS . 1968. Transovarial transmission of the spirochaete Borrelia sogdiana by the tick Ornithodoros papillipes and its effect on biological peculiarities of the pathogen. Parazitologiya 2:193–201.

[17] Geigy R , Aeschlimann A. 1964. Langfristige Beobachtungen über transovarielle Übertragung von Borrelia duttoni durch Ornithodorus moubata. Verlag für Recht und Gesellschaft. 14141443

[18] Burgdorfer W , Varma MG . 1967. Trans-stadial and transovarial development of disease agents in arthropods. Annu Rev Entomol 12:347–376. doi:10.1146/annurev.en.12.010167.002023 5340722

[19] Kitten T , Barbour AG . 1992. The relapsing fever agent Borrelia hermsii has multiple copies of its chromosome and linear plasmids. Genetics 132:311–324. doi:10.1093/genetics/132.2.311 1427031 PMC1205138

[20] Ornstein K , Barbour AG . 2006. A reverse transcriptase–polymerase chain reaction assay of Borrelia burgdorferi 16S rRNA for highly sensitive quantification of pathogen load in a vector. Vector Borne Zoonotic Dis 6:103–112. doi:10.1089/vbz.2006.6.103 16584333

[21] Takacs CN , Wachter J , Xiang Y , Ren Z , Karaboja X , Scott M , Stoner MR , Irnov I , Jannetty N , Rosa PA , Wang X , Jacobs-Wagner C . 2022. Polyploidy, regular patterning of genome copies, and unusual control of DNA partitioning in the Lyme disease spirochete. Nat Commun 13:7173. doi:10.1038/s41467-022-34876-4 36450725 PMC9712426

[22] Williamson BN , Schwan TG . 2018. Conspecific hyperparasitism: an alternative route for Borrelia hermsii transmission by the tick Ornithodoros hermsi. Ticks Tick Borne Dis 9:334–339. doi:10.1016/j.ttbdis.2017.11.009 29174448 PMC5803302

[23] Arakcheeva SG . 1968. [Homovampirism in the tick Alectorobius tholozani and its role in circulation of Borrelia persica in its vectors and in disease foci] in Russian. Works of the Uzbek Scientific Research Institute of Experimental Medical Parasitology & Helminthology 5:245–250.

[24] Beck AF , Holscher KH , Butler JF . 1986. Life cycle of Ornithodoros turicata americanus (Acari: Argasidae) in the laboratory. J Med Entomol 23:313–319. doi:10.1093/jmedent/23.3.313 3735335

[25] Filatov S , Rego R . 2021. Argasidae: Distribution and Vectorial capacity in a changing global environment. CABI International, Wallingford. doi:10.1079/9781789249637.0031

[26] Stearns SC . 1976. Life-history tactics: a review of the ideas. Q Rev Biol 51:3–47. doi:10.1086/409052 778893

[27] Lamattina D , Salomón OD . 2023. Triatoma infestans, to be or not to be autogenic? Acta Trop. 237:106727. doi:10.1016/j.actatropica.2022.106727 36273538

[28] Winston PW , Bates DH . 1960. Saturated solutions for the control of humidity in biological research. Ecology 41:232–237. doi:10.2307/1931961

[29] Folmer O , Black M , Hoeh W , Lutz R , Vrijenhoek R . 1994. DNA primers for amplification of mitochondrial cytochrome c oxidase subunit I from diverse metazoan invertebrates. Mol Marine Biol Biotechnol 3:294–299. 7881515

[30] Krishnavajhala A , Armstrong BA , Lopez JE . 2018. Vector competence of geographical populations of Ornithodoros turicata for the tick-borne relapsing fever spirochete Borrelia turicatae. Appl Environ Microbiol 84:e01505-18. doi:10.1128/AEM.01505-18 30143510 PMC6193384

[31] Lynn GE , Breuner NE , Hojgaard A , Oliver J , Eisen L , Eisen RJ . 2022. A comparison of horizontal and transovarial transmission efficiency of Borrelia miyamotoi by Ixodes scapularis. Ticks Tick Borne Dis 13:102003. doi:10.1016/j.ttbdis.2022.102003 35858517 PMC10880489

[32] RStudio Team U . 2015. Rstudio: integrated development for R. RStudio, Inc, Boston, MA. http://www.rstudio.com.

