# Supplementary figures and images for: Autogenous reproduction by Ornithodoros turicata (Ixodida: Argasidae) females and vertical transmission of the tick-borne pathogen Borrelia turicatae (Spirochaetales: Borreliaceae)
Source: Appl Environ Microbiol. 2023 Oct 25;89(11):e01032-23. doi: 10.1128/aem.01032-23 (PMC10686054; doi:10.1128/aem.01032-23)

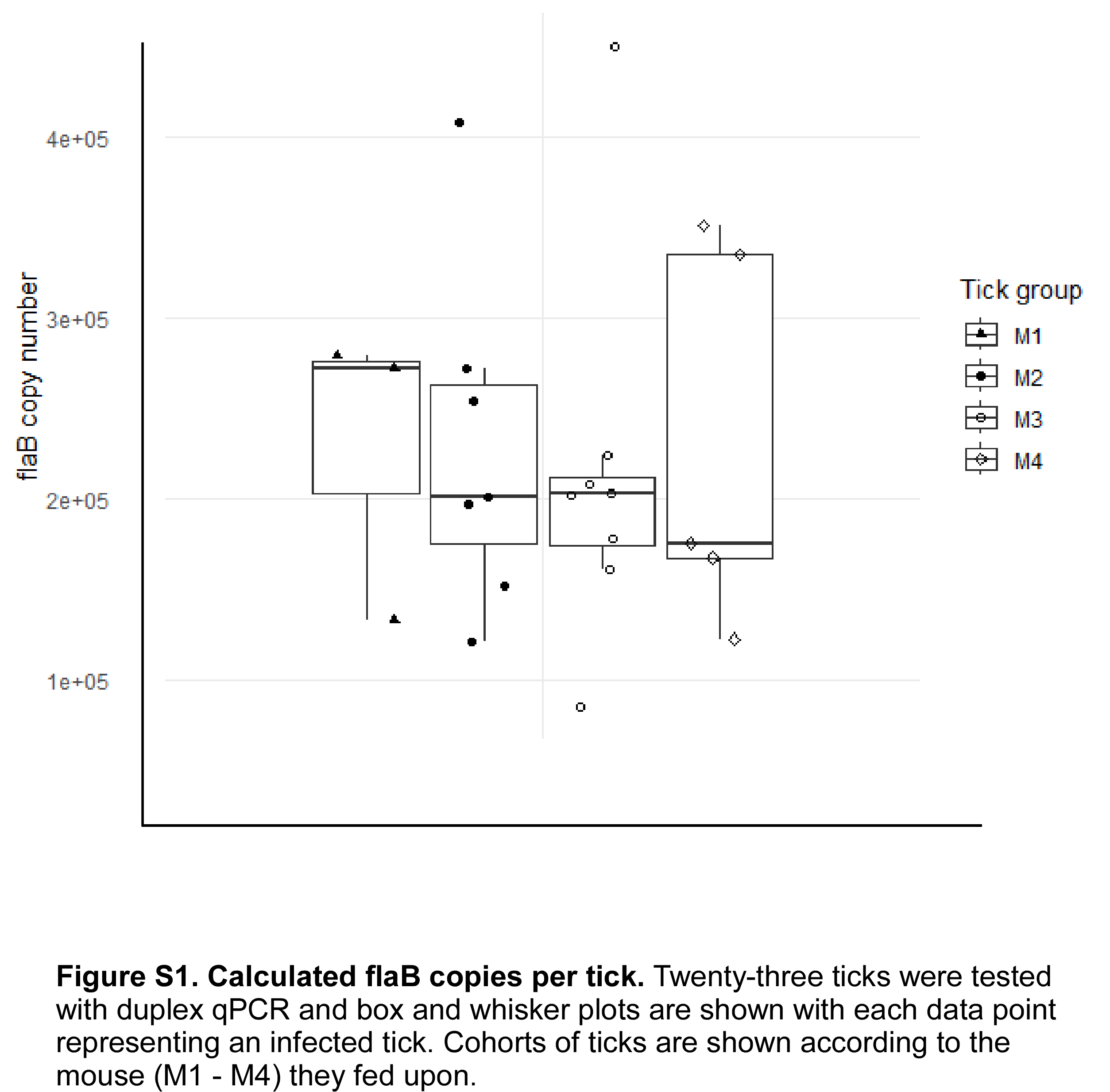

Supplement: Supplemental Figure 1 — Calculated flaB copies per tick. [file aem.01032-23-s0001.tif]
